# Initiation and growth kinetics of solidification cracking during welding of steel

**DOI:** 10.1038/srep40255

**Published:** 2017-01-11

**Authors:** L. Aucott, D. Huang, H. B. Dong, S. W. Wen, J. A. Marsden, A. Rack, A. C. F. Cocks

**Affiliations:** 1 Department of Engineering, University of Leicester, LE1 7RH, UK; 2 Department of Engineering Science, Oxford University, OX1 3PJ, UK; 3 Tata Steel, Research & Development, Swinden Technology Centre, Rotherham, S60 3AR, UK; 4 European Synchrotron Radiation Facility, BP220, F-38043 Grenoble, France

## Abstract

Solidification cracking is a key phenomenon associated with defect formation during welding. To elucidate the failure mechanisms, solidification cracking during arc welding of steel are investigated *in situ* with high-speed, high-energy synchrotron X-ray radiography. Damage initiates at relatively low true strain of about 3.1% in the form of micro-cavities at the weld subsurface where peak volumetric strain and triaxiality are localised. The initial micro-cavities, with sizes from 10 × 10^−6^ m to 27 × 10^−6 ^m, are mostly formed in isolation as revealed by synchrotron X-ray micro-tomography. The growth of micro-cavities is driven by increasing strain induced to the solidifying steel. Cavities grow through coalescence of micro-cavities to form micro-cracks first and then through the propagation of micro-cracks. Cracks propagate from the core of the weld towards the free surface along the solidifying grain boundaries at a speed of 2–3 × 10^−3^ m s^−1^.

Welding is the most effective way to join metals permanently, and it is estimated that over 50% of global domestic and engineering products contain welded joints. In welding, work-pieces are mixed with filler materials and melted, to form a pool of metal that upon solidification becomes a strong, permanent joint. Cracking may occur during solidification of the melt pool, and solidification cracking is an important issue in welding, casting and solidification-related additive manufacturing processes. If undetected, the cracking defects can act as stress concentration sites which could lead to premature failure via fatigue, as well as offer favourable sites for hydrogen assisted cracking^1^,^2^,^3^ and stress corrosion cracking^4^. Because of this, solidification cracking defects are widely studied and have been the subject of investigation for several decades^4^,^5^,^6^,^7^,^8^,^9^,^10^,^11^,^12^,^13^,^14^,^15^. A sustained deficiency in understanding the fundamental mechanism for damage has driven experimental efforts towards the observation of the phenomenon *in situ*.

Early *in situ* experiments were focussed on favourable organic alloys because of the technical problems associated with metallic alloys (namely the metal opacity and density). Farup *et al*.^16^ successfully observed solidification cracking *in situ* in a directionally solidified succinonitrile-acetone alloy using a Bridgman type experiment. The method was later refined by Grasso *et al*.^17^ and damage was observed at grain boundaries in organic alloys. *In situ* observations of solidification cracking in metallic materials derived by the development of third generation synchrotron sources. Phillion *et al*.^18^,^19^ used a synchrotron micro-tomography approach to image the three dimensional morphology of solidification cracks in aluminium alloys. The results showed that porosity in the alloy acts as pre-existing nuclei for damage growth under semi-solid deformation. The author later employed a radiography approach^20^ to observe semi-solid deformation and failure. A similar approach was then employed by Aveson *et al*.^21^, although at a much smaller scale, to observe damage initiation and propagation, at the dendritic scale, in an Al-Sn binary alloy. Gourlay *et al*.^22^ and Fonseca^23^ demonstrated the existence and importance of granular behaviour in aluminium alloys during solidification. Vernede, Rappaz, and co-workers then adopted the granular simulation method for discrete element simulations of solidification cracking^24^,^25^,^26^. Puncreobutr *et al*.^27^,^28^,^29^ highlighted the importance of second phase inter-metallic’s on the solidification cracking of Al-Si-Cu alloys. Recently Kareh *et al*.^30^ Karagadde *et al*.^31^ utilised time-resolved tomography to reveal rotation and transgranular liquation during semi-solid processing. Wang *et al*.^32^ observed refinement and growth enhancement in Al-Cu alloys under magnetic field solidification. The studies show that solidification cracking is a highly complex phenomenon which involves heat flow, fluid flow and various other factors such as material chemistry and processing parameters.

The remaining challenge lies in elucidating the failure mechanism during high-solidification-rate manufacturing processes, such as welding and additive manufacturing of industrially relevant materials, for instance, commercial steel. To address this, in current research, a novel strain-based deformation stage has been developed and embedded into the European Synchrotron Radiation Facilities (ESRF) high resolution imaging beamline, ID19. The test rig has been dubbed the ADW test rig. The beamline is installed on a low-beta section of the storage ring. ID19 has a small source size while the divergence and the length of 145 m allows one to operate macroscopically large beam diameters at the position of the experimental hutch. Furthermore, the length of ID19 reduces the effective source size contribution to the images and therefore allows for the coherence properties of the beam to be exploited by means of inline X-ray phase contrast^33^. Solidification cracking during welding of steel is observed *in situ* using high-speed, high-energy radiography. Synchrotron X-ray micro-tomography is implemented to rebuild and analyse the 3D crack network. Damage initiation and growth kinetics are studied and a propagation growth mechanism controlled by the advancing columnar dendrites of the solidifying weld pool is elucidated for solidification cracking during high-solidification-rate processing of steel.

## Results

### *In situ* radiography of solidification cracking during welding

**Figure 1:**
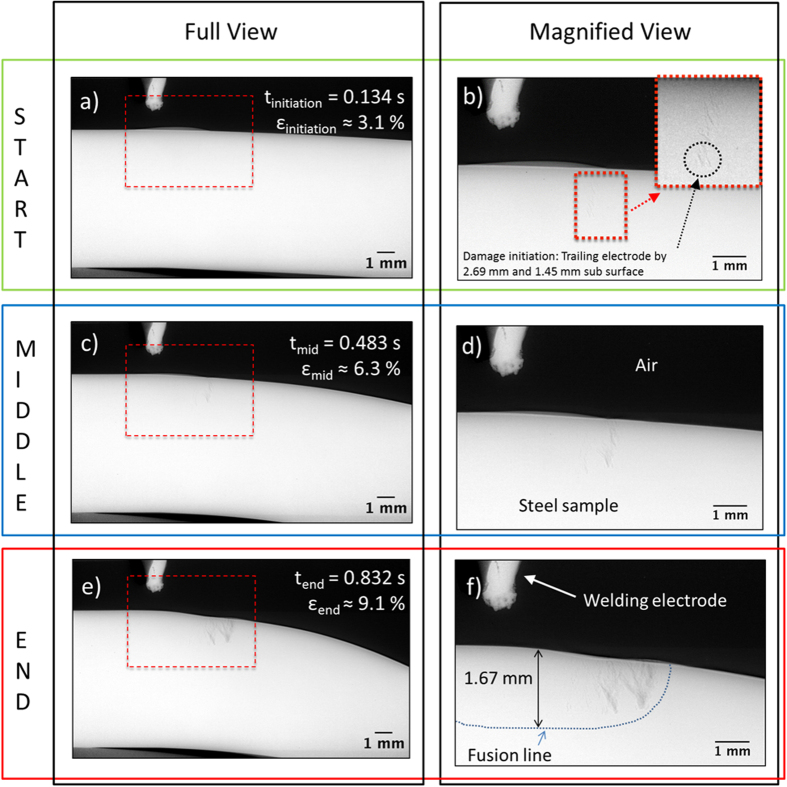
Results of *in situ* radiography analysis, showing (**a**,**b**) the initiation of solidification crack at 0.134 s of bending with a true bending strain of 3.1%. Crack initiates trailing the welding electrode by 2.69 × 10^−3^ m at 1.45 × 10^−3^ m sub-surface, (**c**,**d**) crack observed at mid-point of test, (**e**,**f**) the end of solidification cracking at 0.832 s of bending and a true bending strain of 9.1%.

Figure 1 displays a series of processed radiographs. As shown in the Figure, the white tip in the top left of the images is the tungsten welding electrode. The large white part in the centre of the images is an EN1A test sample. Figure 1(a) highlights the point at which damage initiation was observed in the radiography sequence. The weld solidification cracks are easily distinguishable, being darker than the bulk sample. This is due in part to a lower attenuation through the cracking interface. However, the main source of the interface clarity can be attributed to the refraction based coherent imaging technique and wide propagation distance between the sample and detector.

**Figure 2:**
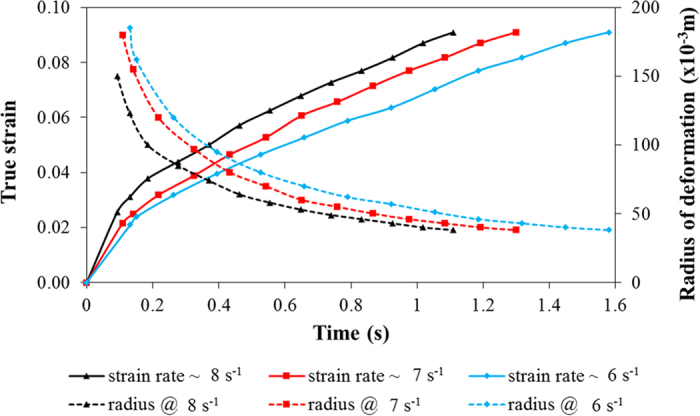
*In situ* measurement of deformation radius and true strain during testing.

*In situ* measurements of deformation radius and true strain are presented in Fig. 2. Cracks appear after 0.134 s of bending under a loading rate of 0.135 m s^−1^. The true strain for damage initiation is 3.1%. Detailed analysis of the damage shown in Fig. 1(c) reveals that the solidification cracks appear to initiate trailing the welding electrode by 2.69 × 10^−3^ m at 1.45 × 10^−3^ m sub-surface, approximately 0.22 × 10^−3 ^m behind the solidification front within the weld.

Upon further loading, the cracks propagate vertically and penetrate the upper surface of the sample after 0.324 s. The velocity of the fracture is measured at approximately 2.2 × 10^−3 ^m s^−1^ in the 2D image plane. However, due to the 3D nature of the cracking, this could equate to speeds of up to 3.2 × 10^−3^ m s^−1^ when considering propagation in a plane inclined at an angle up to 45° to the 2D image plane.

Analysis of radiographs reveals that after the initial damage has penetrated the surface, further cracking then begins to initiate sub-surface in the region to the left of the initial initiation site (closer to the heat source) under the continued influence of the augment bending strain and follow a similar vertical propagation path. This time however, it appears that the cracks also begin to converge laterally with the initial crack. Damage continues to develop in this manner until 0.832 s of bending (Fig. 1b). Damage initiation and growth then ceases as the increase of the augmented strain diminishes, with the total observed cracking displayed in Fig. 1(f) at 9.1% true strain. From the analysis it is possible to conclude that damage occurred in the EN1A sample (tested at a 0.135 m s^−1^ deformation rate) over a 0.698 s time period.

### Tomographic analysis of the solidification crack network

**Figure 3:**
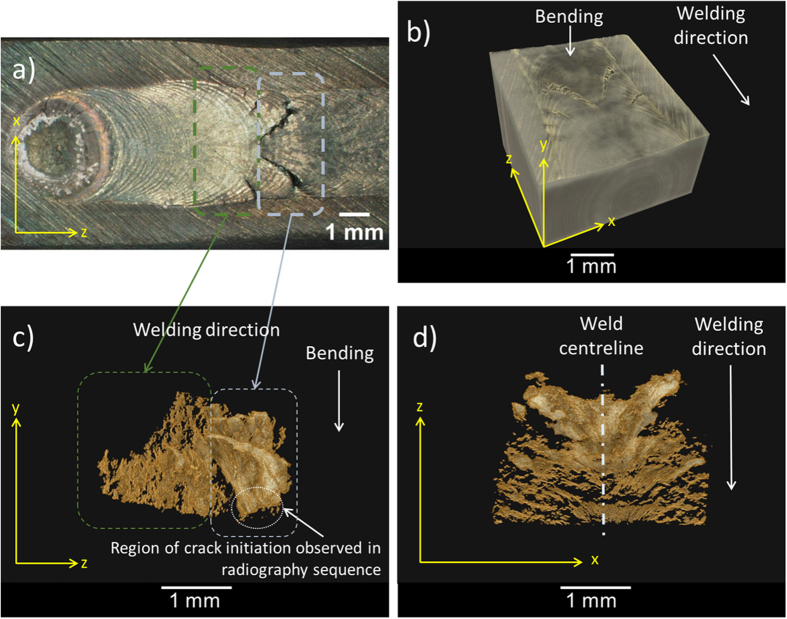
3D tomographic reconstruction and characterisation of weld solidification cracks, showing: (**a**) macrograph of the sample weld containing solidification cracks, (**b**) 3D reconstruction of the crack containing volume. The weld and some solidification cracks are visible on the top surface. The yellow arrows denote the Cartesian coordinate system for view plane reference, (**c**) Y-Z plane 2D view of the solidification crack network. The blue box represents a “mature” solidification crack that is fully grown. The red box represents “infant” solidification cracks that are primarily sub-surface and invisible from visual inspection of Fig. 3(a,d) X-Z plane view of the solidification crack network showing somewhat symmetric features in terms of the angle of cracking either side of the weld centreline due to the symmetrical nature of the weld itself.

Figure 3 displays a typical 3D tomographic reconstruction of the solidification crack network in the TIG weld of the EN1A steel sample. The morphology of the cracks appears to be associated with the profile of the weld pool, as the network resembles arches that loop from the lateral edges of the weld around the semi-circle weld geometry. The network has been further divided into two regions depicted by the broken line boxes in Fig. 3(a and c). The blue box represents a “mature” solidification crack that is fully grown. The “mature” crack makes up the predominant volume of total cracking (~75%) and propagates through the largest area on sample surface. It is essentially one crack that contains the sites at which the solidification cracks first initiated and subsequently grew and coalesced. The mature crack and initiation sites will be examined in the damage initiation sub-section. The red box represents “infant” solidification cracks that are primarily sub-surface and invisible from visual inspection of Fig. 3(a). These cracks initiate at a later stage in the *in situ* radiography sequence presented in Fig. 1. As such, they do not have sufficient time and applied strain to grow to a “mature” stage whereby they break through the surface. There are many isolated as well as interlinked cracks in this region. The morphology of the network in the “infant” region can give an insight to the morphology of the “mature” crack during early stage development, and will be discussed further in the damage growth sub-section.

## Discussion

### Damage initiation

**Figure 4:**
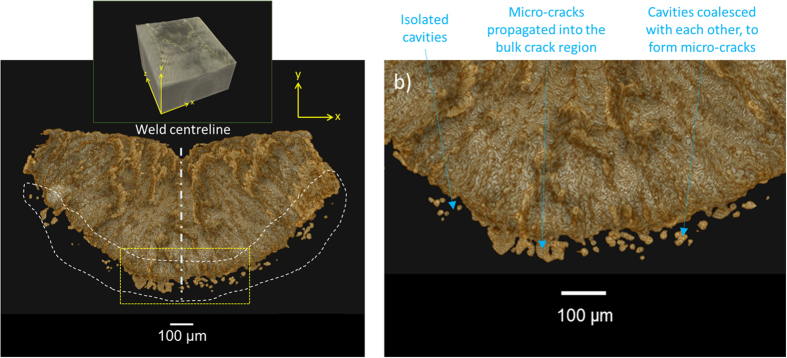
3D tomographic reconstruction of fracture initiation sites. Image extracted from Fig. 3 on the X-Y plane, approximately 2.7 × 10^−3^ m from the weld end in the Z direction, (**a**). The yellow broken square highlights the fracture initiation sites as observed in the *in situ* synchrotron X-ray radiography sequence (shown in Fig. 1a and b), which is observed at a higher magnification in (Fig. 4b). The white broken lines highlight a series of isolated and coalesced micro-cavities. Some of the coalesced micro-cavities, termed as micro-cracks in the current study, are formed away from the bulk crack region, whereas others have propagated further to join the bulk crack region, as detailed in (Fig. 4b).

The damage initiation sites, observed during the *in situ* synchrotron X-ray radiography in Fig. 1(a and b), has been located on the 3D volume reconstructed from the synchrotron X-ray tomographic data and presented in Fig. 4. Isolated micro-cavities are situated away from the bulk crack, inherent to the region where damage is observed to initiate during *in situ* radiography.

**Figure 5:**
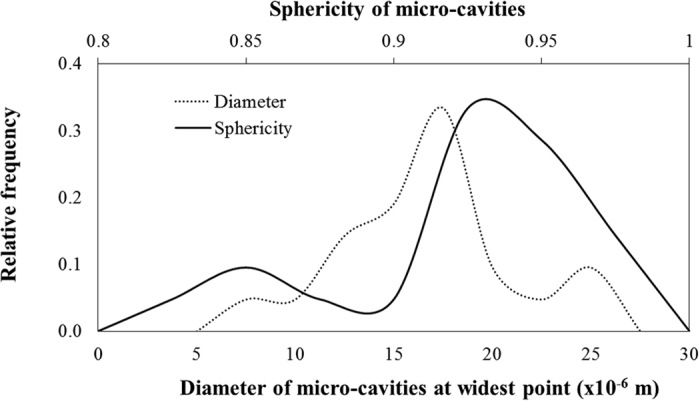
Quantitative analysis of the diameter and sphericity of isolated micro-cavities identified in Fig. 4(b).

Examination of the micro-cavities in Fig. 4(b) reveals that there are 21 isolated cavities. Quantitative analysis of these isolated cavities, presented in Fig. 5, reveals a size range between 10–27 × 10^−6^ m (mean average = 18 × 10^−6^ m) with highly spherical morphologies between 0.81–0.96 sphericity (mean average = 0.91). In some cases, the micro-cavities coalesce with each other to form isolated micro-cracks, indicating that micro-cavity coalescence is the dominant mechanism for growth in the early stages of damage development.

**Figure 6:**
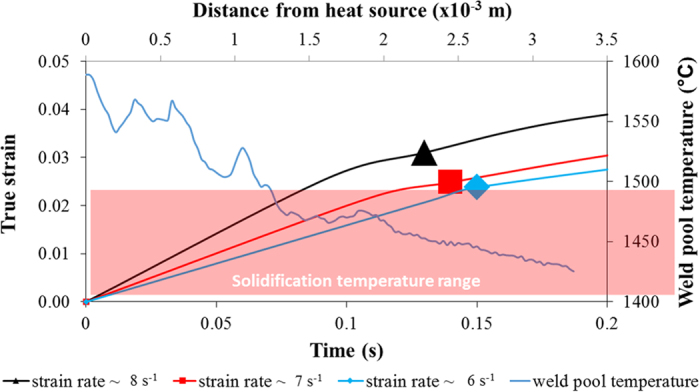
Calculation of true bending strain and measurement of weld pool temperature as a function of time. The resultant true strain curves at different strain rates and weld pool temperature history profile are used to quantify kinetics for damage initiation. The three markers indicate damage initiation strain calculated from the image sequence of *in situ* radiography at 8 s^−1^, 7 s^−1^ and 6 s^−1^ strain rate respectively.

Temporal evolution of true strain and weld pool temperature, calculated from the *in situ* synchrotron X-ray radiography sequence and thermocouple recordings respectively, are used to quantify the kinetics of damage initiation. The results, given in Fig. 6, reveal that damage initiation is dependent upon strain rate. As strain rate decreases, damage initiates at a later stage and under relatively lower true strain. For instance, damage initiates in the sample tested at ~8 s^−1^ strain rate after 0.13 s under 3.1% true strain. In the sample tested at ~6 s^−1^ strain rate, damage initiates after 0.15 s under 2.4% true strain.

Assuming a consistent solidification rate between tests, this equates to damage initiation temperatures of 1453 °C and 1445 °C, respectively. To relate these to the corresponding semi-solid microstructure, the damage initiation temperatures are assessed against volume fraction of liquid through the solidification range. Based on the chemistry specified in the methods, Thermo-Calc simulations under equilibrium conditions show that at 1453 °C the volume fraction of liquid in the weld pool would be 0.26 and at 1445 °C the volume fraction of liquid would be 0.16. It has to be stated that these volume fraction of liquid values are only indicative and for illustrative purposes only since the weld pool solidification-rate is high and not an equilibrium phenomenon. However it does prospectively indicate that higher strain rates would induce cracking at a relatively higher volume fraction of liquid. The true strain required to initiate the damage in this thermodynamic state is higher than for low strain rate due to the increased presence of liquid in the semi-solid skeleton. The extra liquid maintains permeability within the semi-solid skeleton, allowing liquid metal to remain mobile and heal any deformation-induced cavity openings. As a result, the strain required for damage initiation increases.

It is evident, in the early stages, that solidification cracking is controlled by the formation and growth of internal micro-cavities or voids. The process of void formation leading to solidification cracking is complex and poorly understood. Farup *et al*.^22^ tried to identify the underlying mechanisms of damage initiation and observed three different mechanisms for solidification crack nucleation: (1) directly as elongated pores or tears, (2) on pores caused by solidification shrinkage, or (3) as round pores nucleated in the liquid constituting a healed hot tear. Campbell^34^ proposed that voids form via separation of the solid-liquid interface and at entrained oxides or other heterogeneous nuclei as the cavitation pressure required to form voids is large in comparison to the expected shrinkage pressure drop during solidification. Previous *in situ* experiments^20^,^35^,^36^, with finer resolutions and smaller gauge volumes, show that semi-solid tensile deformation promotes the flow of liquid metal to strain-localised regions. The resultant concentration of liquid metal in the strain-localised region allowed the semi-solid to accommodate large true strains (~14%) prior to damage^29^. With increasing strain, liquid feeding becomes insufficient and cavities first form in the sample core where strain and triaxiality are greatest^37^,^38^,^39^. This is consistent with analysis presented in this study.

The initial size of the nucleating cavities and their relationship to the semi-solid microstructure and stress-state are important for understanding the underlying mechanisms of solidification crack nucleation. In this study, all of these important aspects have been identified and quantified. The micro-cavities manifest at relatively low true strain (~3.1%) when compared to damage initiation strains reported in previous studies (e.g. ~14%^35^). The reasoning for this can be explained as follows: firstly, columnar grain structures are consistently observed in all test samples presented within this manuscript. The grain structure near the fusion line of a weld is dominated by epitaxial growth when the base metal and the weld metal have the same crystal structure. Away from the fusion line, however, the grain structure is dominated by a different mechanism known as competitive growth.

During solidification grains tend to grow in the direction perpendicular to fusion boundary, because this is the direction of the maximum temperature gradient and hence maximum heat extraction. Columnar dendrites tend to grow from the fusion boundary towards the centre line of the weld pool under high thermal gradient. This mechanism of competitive growth dominates the grain structure of the bulk weld metal and results in a columnar grain structure. Columnar grains are inherently less permeable than equiaxed grains studied in the previous work. Lower permeability of the mush will hinder liquid accumulation in the strain-localised region, reducing the amount of strain that can be accommodated, and therefore promoting damage initiation at relatively low strain. Secondly, previous studies were also carried out with either isothermal or slow-solidification-rate experiments. Under isothermal conditions, the damage initiation strain would vary as a function of test temperature (fraction solid/liquid). As such, results of such tests can only give an accurate representation of damage at one thermal instance in the solidification process, whereas in reality weld solidification cracking will occur across a range of temperature and strain conditions due to the transient nature of the welding process. It is also important to appreciate that different mechanisms could also be active at different stages of damage development. Damage initiation kinetics reported in the current study represents real conditions exhibited in TIG welding which is classified as a high-solidification-rate manufacturing process along with other welding and additive manufacturing processes. Further work is necessary to identify the specific features and chemistry at the initiation sites which could be particularly associated with micro-cavity nucleation.

### Damage growth

**Figure 7:**
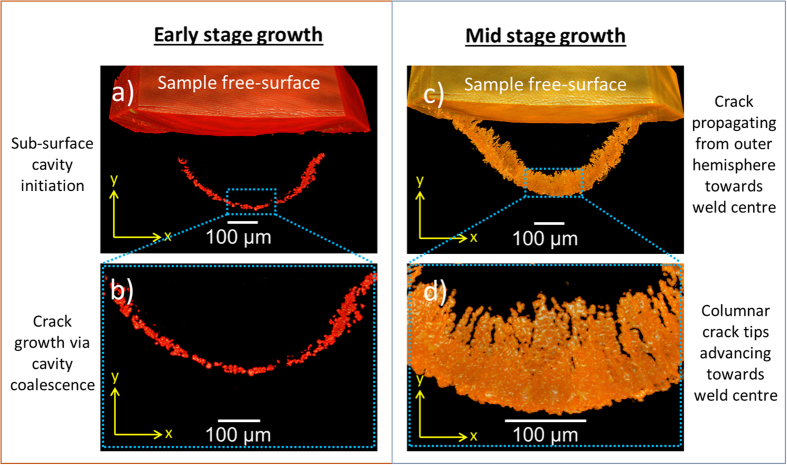
Transverse cross-sectional tomography images showing solidification crack growth path. Cross-sections extracted from the region depicted by the broken red box in Fig. 3(a and c) at distance 1.6 × 10^−3^ m (Figure a and b) and 1.8 × 10^−3^ m, (Figure c and d) from the weld end.

A propagation growth mechanism controlled by the advancing columnar dendrites of the solidifying weld pool has been elucidated from micro-tomography analysis. Transverse cross-sectional images at various stages of growth are extracted from the 3D crack network displayed in Fig. 3(c). Images of the infant stages of crack growth are extracted from the region depicted by the broken red box in Fig. 3(a and c) and presented in Fig. 7. In the initial stage of growth, displayed in Fig. 7(a), micro-cavities with average sizes of ~18 × 10^−6^ m and high degrees of sphericity (~0.91) have formed in the weld sub-surface directly underneath the solidifying weld pool. The micro-cavities manifest around the hemisphere of the weld bead to form an arch like structure. Closer examination of the region in Fig. 7(b) reveals that these micro-cavities coalesce to form micro-cracks that vary in size and morphology. At later stages of growth, depicted in Fig. 7(c), a full arch is formed and the sample surface is penetrated on both sides of the weld. The damage grows at a fairly even rate inwards, towards the weld centre and sample free-surface. The kinetics of damage growth towards the free-surface from the sample core is in agreement with similar studies on binary Al-Cu^20^,^35^, Al-Si-Cu^29^, and other commercial alloys^18^. However, this is the first time that the damage growth kinetics are reported for high-solidification-rate processing of steel such as TIG welding. In contrast, Terzi *et al*.^36^ observed qualitatively that the damage grew in an Al-Cu alloy from pre-existing pores on the sample free-surface towards the core. The reasoning for this particular damage growth mechanism can be attributed to granular behaviour, with the grains rotating and opening gaps at the surface^40^. Hence, the existing pores at the surface play a bigger role with respect to damage development. The granular behaviour occurs due to a small gauge diameter (1.5 mm) and large grain size. Insufficient numbers of grains across the specimen’s diameter may not be able to represent continuum behaviour of the material.

Closer examination of the crack network in Fig. 7(d) reveals several columnar crack tips that are advancing towards the weld centre. This hints towards a propagation growth mechanism controlled by the advancing columnar dendrites of the solidifying weld pool.

Further work is needed to identify the thermodynamic state and localised structural features at micro-cavity initiation sites and fracture surfaces. The results of such work will facilitate an elaboration to the mechanism proposed in this study.

## Methods

### Materials and test procedure

Three days beam time were awarded by the ESRF for this research. During the experiments, a total of twenty seven samples were tested. Samples were taken from three steel variants and tested at three different strain rates. Each test was then repeated three times to demonstrate repeatability between tests.

In this article, the results from EN1A mild steel are presented. Test samples of 8 × 8 × 300 × 10^−9 ^m^3^ were prepared from the mild steel with a nominal chemistry (wt%) of 0.15C-1Mn-0.35Si-0.06P-0.6 S. To improve X-ray image quality, it is imperative to minimise the path length through the sample. The 8 × 10^−3 ^m sample thickness was selected as it was the smallest feasible to weld upon. For the welding, a high-solidification-rate tungsten-inert-gas (TIG) process was used. TIG welding was carried out at 10 V and 98 A with a non-consumable tungsten electrode in DC-ve polarity. During welding, weld pool solidification temperature was measured by tungsten-rhenium Type C thermocouples. The resultant temperature curve was used in conjunction with thermodynamic predictions of the solidification temperature range to give a crude measure of solidification rate. The solidification temperature range, determined by Thermo-Calc simulations (equilibrium conditions) is between 1403–1495 °C (92 °C) and the time taken for the weld pool to cool through this temperature range is 0.18 s. As such the solidification rate is estimated at 511 °C s^−1^.

**Figure 8:**
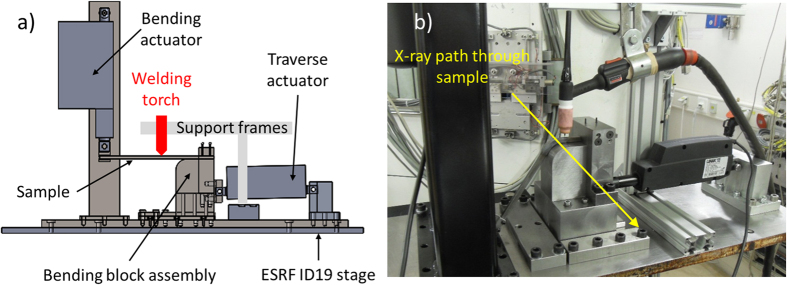
ADW test rig setup showing, (**a**) Side view of the design model in pre-test settings schematically illustrating key components, (**b**) ADW test rig embedded into ID19 beamline. The sample is in post-test deformed state and the yellow arrow dictates the approximate path of the X-ray through the 8 × 10^−3^ m thickness sample towards the detector.

Figure 8(a) shows the side view from the CAD model of the ADW test rig at the starting position of the test. Figure 8(b) is a photograph of the experimental setup embedded into ID19 experimental hutch after being fully tested offline. The equipment is designed to carry out stand-alone small-scale Varestraint (variable-restraint) weldability tests, while also complying for integration to the ESRF ID19 experimental hutch.

Prior to the test, the sample is locked into the clamping device on top of the bending block with a 30 × 10^−3^ m grip to attach the earth clamp for welding. The bending block assembly is attached to one of the two electric actuators powered by a 12 V battery (traverse actuator, Fig. 8a). The horizontally orientated traverse actuator (Linak LA12) supplies a 750 N load at speeds of up to 14 × 10^−3 ^m s^−1^ across a 40 × 10^−3 ^m stroke length. This actuator traverses the bending block assembly along the table to create the motion to weld a 40 × 10^−3^ m length along the sample. Guide rails located on the surface of the base table interlink with the bending block assembly to ensure a straight travel path, normal to the beam.

The opposite side of the test sample is then attached to the second actuator (bending actuator, Fig. 8(a), Linak LA36) with a vertical orientation. This provides a 500 N load at speeds of up to 135 × 10^−3 ^m s^−1^ across a 150 × 10^−3 ^m stroke length. This actuator bends the sample during welding. Bending causes the sample to deform around the radius (60 × 10^−3 ^m) of the bending block and induces an augmented strain to the sample to stimulate solidification cracking.

For the experiment, welding velocity was controlled at 2.5 × 10^−3^ m s^−1^. The bending load was initiated during welding at t = 14 s (approx. 35 × 10^−3 ^m weld length). After bending was completed (approx. 1 second later), the welding arc was turned off, but the traverse continued for a further ~1 second until the full 40 × 10^−3 ^m stroke length was reached. Bending speed was assessed at three different increments; 100%, 85%, and 70% of LA36 full speed. Assuming strain is applied linearly, this equates to approximate true strain rates of 8, 7, and 6 s^−1^ respectively.

### *In situ* synchrotron X-ray radiography

In order to reach a sufficiently high photon flux density at the desired X-ray energy of around 110 keV, ID19 was operated in white beam mode for radiography. The light of the beamline’s wiggler (gap 45) was filtered by a diamond window, 5.6 × 10^−3^ m Al and 6 × 10^−3^ m Cu. The resulting bandwidth is comparably large (ΔE/E = 90%, peak around 117 keV) but allows for radiography imaging at frame rates up to 1 kHz. The resultant photon flux density was approximately 7 × 10^11^ photons mm^−1^s^−1^. The corresponding indirect detector consisted of two identical objectives in tandem geometry (Hasselblad, type: HC 2.2/100 MM) leading to an effective 1:1 magnification: the lens-system projects the luminescence image of a 750 × 10^−6^ m thick LuAG:Ce single-crystal scintillator (Crytur, Czech Republic) onto the sensor of a camera. A periscope-like design with a folded optical path via a mirror is utilised in order to keep the objectives and electronics out of the intense hard X-ray photon beam. A pco.dimax camera was chosen (PCO AG, Germany; 2016 × 2016 pixels, 11 × 10^−6^ m pixel size, 50% peak quantum efficiency at 500 × 10^−9^ m, 36 GB on-board memory for fast intermediate storage, 1279 full images per second (fps) maximum frame rate). For the imaging, a view window of 1872 × 1000 pixels was employed with a 10 × 10^−3^ m/pixel resolution. The camera recorded continuously images at a rate of 1000 fps to satisfy the temporal demands in observing the solidification cracking *in situ*. In order to enhance the contrast of the cracks by means of X-ray inline phase contrast, a propagation distance of 7.3 m between sample and detector was realised^41^.

All post-processing of the captured image data was performed using the public domain Java image processing program ImageJ^42^, with the assistance of a simple processing routine. Firstly a “median Z stack” was made from a 500 frames of a reference image (*I*_*0*_) without the sample and equipment present in the field of view. This stage is implemented to average out the high contrast noise speckles. Next, the raw data *(I)* was normalised against the reference image by a -log(*I/I*_*0*_) math function. This stage effectively masks the background noise created by the beam to focus on the test specimen. True strain, *ε*_*true*_, in bending is calculated by:


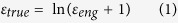


where, *ε*_*eng,*_ is the nominal engineering strain of the bend, given by:


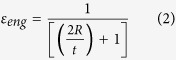


Strain rate, 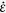, is assumed to be linear and defined by:


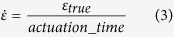


where, *t* is the initial material thickness and *R* is the inner bend radius of the deformed sample. This allows for strain calculations to be made *in situ* by measuring radius of deformation, observed in radiographs, at any given interval during deformation.

### Synchrotron X-ray micro-tomography

Micro-tomography was carried out post-mortem with a similar experimental configuration to that described in the previous section. The insertion device settings and filters remained the same. Experiments were carried out while the ESRF was operated in so-called 4bunch operation, resulting in a reduced photon flux which is still sufficient for the comparable long scans of the static samples. A FReLoN CCD camera (type: 2k/F_A7899, ESRF in-house development, 2048 × 2048 pixels, 14 × 10^−6^ m pixel size) detector was combined with 4:1 magnification optics resulting in an effective pixel size of 3.5 × 10^−6^ m. As scintillator a 250 × 10^−6^ m LuAG:Ce was chosen. The propagation distance was 1.2 m. 1200 projection images were recorded per sample in order to ensure good tomographic reconstruction quality. For 3D image reconstruction the ESRF in-house software PyHST_2 was used which is based on the filtered-back projection approach. The resultant image stacks were then filtered using a 3D median filter to remove high contrast speckle noise, and then sharpened to enhance the edges using ImageJ. Volume rendering of the stacks and volumetric analysis was carried out in Drishti volume exploration software. Cavity sphericity was calculated using:


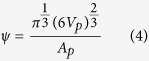


where, *V*_*p*_ is the cavity volume and *A*_*p*_is the cavity surface area. Both features were measured from analysis in the volume editing software and used as input in Eq. 4.

## Additional Information

**How to cite this article:** Aucott, L. *et al*. Initiation and growth kinetics of solidification cracking during welding of steel. *Sci. Rep.* **7**, 40255; doi: 10.1038/srep40255 (2017).

**Publisher's note:** Springer Nature remains neutral with regard to jurisdictional claims in published maps and institutional affiliations.
